# Subcellular localisations of the CPTI collection of YFP-tagged proteins in *Drosophila* embryos

**DOI:** 10.1242/dev.111310

**Published:** 2014-10

**Authors:** Claire M. Lye, Huw W. Naylor, Bénédicte Sanson

**Affiliations:** The Department of Physiology, Development and Neuroscience, University of Cambridge, Downing Street, Cambridge CB2 3DY, UK

**Keywords:** GFP, Epithelium, Morphogenesis, Protein trap

## Abstract

A key challenge in the post-genomic area is to identify the function of the genes discovered, with many still uncharacterised in all metazoans. A first step is transcription pattern characterisation, for which we now have near whole-genome coverage in *Drosophila*. However, we have much more limited information about the expression and subcellular localisation of the corresponding proteins. The Cambridge Protein Trap Consortium generated, via *piggyBac* transposition, over 600 novel YFP-trap proteins tagging just under 400 *Drosophila* loci. Here, we characterise the subcellular localisations and expression patterns of these insertions, called the CPTI lines, in *Drosophila* embryos. We have systematically analysed subcellular localisations at cellularisation (stage 5) and recorded expression patterns at stage 5, at mid-embryogenesis (stage 11) and at late embryogenesis (stages 15-17). At stage 5, 31% of the nuclear lines (41) and 26% of the cytoplasmic lines (67) show discrete localisations that provide clues on the function of the protein and markers for organelles or regions, including nucleoli, the nuclear envelope, nuclear speckles, centrosomes, mitochondria, the endoplasmic reticulum, Golgi, lysosomes and peroxisomes. We characterised the membranous/cortical lines (102) throughout stage 5 to 10 during epithelial morphogenesis, documenting their apico-basal position and identifying those secreted in the extracellular space. We identified the tricellular vertices as a specialized membrane domain marked by the integral membrane protein Sidekick. Finally, we categorised the localisation of the membranous/cortical proteins during cytokinesis.

## INTRODUCTION

Fluorescent proteins have revolutionised our ability to observe proteins in live tissues. Single gene studies now routinely ectopically express fluorescently tagged versions of a given protein to identify its localisation. Beyond a single gene approach, efforts have been made in the past decade to fluorescently tag many proteins in parallel. In yeast, it was possible to tag 75% of endogenous proteins by inserting a GFP exon at the 3′ end of open reading frames (Huh et al., 2003). Partly because of the lack of efficient homologous recombination, this feat is difficult to replicate in multicellular organisms, so large-scale production of fluorescently tagged proteins relies instead on transposon-mediated tagging. Transposons are modified to integrate an exon encoding a fluorescent protein at near-random locations into the genome. When the transposon inserts within an intron, this can result in a tagged protein expressed from its natural promoters.

Screens for protein traps using the mobilisation of fluorescent exons were first carried out in *Drosophila* (Morin et al., 2001; Clyne et al., 2003; Buszczak et al., 2007; Quinones-Coello et al., 2007). These screens recovered both enhancer trap and protein trap lines, because the main transposable element used, the P-element, is biased towards insertion in sequences 5′ to coding sequences. From these studies, over 449 true protein trap lines were generated, corresponding to the in-frame tagging of 226 unique genes with GFP (Aleksic et al., 2009). Outside *Drosophila*, large-scale tagging of full-length proteins with fluorescent exons to analyse subcellular localisation is developed in vertebrates such as zebrafish (Trinh and Fraser, 2013) and in the model plant *Arabidopsis* (Tanz et al., 2013).

The accompanying paper reports the generation in *Drosophila*, of a collection of proteins tagged with YFP using new vectors based on *piggyBac* transposition to principally produce protein traps (Lowe et al., 2014). This new collection is composed of over 600 Cambridge Protein Trap Insertion (CPTI) lines, corresponding to just under 400 identified genes. The subcellular localisations of the CPTI lines have been characterised in many tissues by a consortium of UK groups and the information is centralized in the Flyprot website, www.flyprot.org (Lowe et al., 2014). In this paper, we aim to provide a further resource to the community by characterising the subcellular localisation of the complete CPTI collection of YFP-trap proteins in live *Drosophila* embryos. We had two main goals: to give clues to the function of uncharacterised proteins and to identify markers for organelles and subcellular regions. Such markers are still scarce in *Drosophila* but are crucial to conducting cell biology studies in live tissues, embryonic or other.

To characterise the subcellular localisations, we imaged cellularising embryos (stage 5) because the cells are regularly arranged and larger than at other stages of development (Mazumdar and Mazumdar, 2002; Lecuit, 2004). For the protein traps localising at the plasma membrane or cortex, we expanded our characterisation to stages 6 to 10, to include epithelial morphogenesis during axis extension and early segmentation (Lye and Sanson, 2011). Because the tagged proteins are expressed at endogenous levels, we used spinning disk confocal microscopy coupled with an EM-CCD camera to increase the sensitivity of detection. This paper systematically identifies the subcellular localisation of hundreds of *Drosophila* proteins and provides a comprehensive resource for cell biology studies.

## RESULTS

### Overview of the expression and subcellular localisation of the CPTI lines

**Fig. 1.**
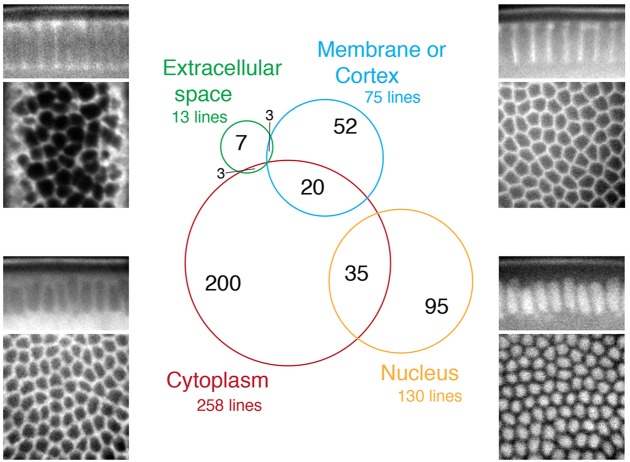
**Subcellular localisation of the CPTI collection at cellularisation.** Four-hundred and fifteen YFP-tagged lines are expressed at stage 5 during cellularisation. Nuclear, cytoplasmic, membranous or extracellular subcellular localisations of the proteins were identified at this stage by examining confocal sections showing the arrangement of cells in the apico-basal plane (top panels) and in the plane of the tissue (bottom panels). The Venn diagram shows the number of lines localising in a given compartment, including those detected in two compartments.

Out of 560 lines screened, 415 lines (74%) were expressed at stage 5 (cellularisation), 507 (91%) at stage 11 (mid-embryogenesis) and 521 (93%) at stage 15 and later (late embryogenesis) (supplementary material Table S1). Most of the lines are expressed in all tissues without obvious patterns at stage 5 and 11. The main exception are lines showing metameric patterns: at stage 5, two insertions in the Teneurin homologue Ten-m are expressed in stripes (supplementary material Fig. S1A); at stage 11, 31 lines show a metameric pattern, including genes known to be segmentally expressed such as: *frizzled 2*, the glypicans *dally* and *dally-like*, *semaphorins 1b* and *2a* and *netrin A* and *B*, and genes for which this was previously unknown such as *arginine kinase* (supplementary material Fig. S1B). At stage 15 or later, when the larval organs have formed, we found more patterns (supplementary material Fig. S1D-H), the most frequent being expression in the central nervous system (137 lines, 26%, supplementary material Table S1), but here again the tagged proteins are in majority expressed in most tissues. All expression pattern information is summarised in supplementary material Table S1 and some notable patterns are shown in supplementary material Fig. S1 and the accompanying paper (Lowe et al., 2014). We focused on the 415 lines showing expression at stage 5 to determine their subcellular localisation and found that 258 YFP-trap proteins localised in the cytoplasm, 130 in the nucleus, 75 at the membrane and/or cortex and 13 in the extracellular space (Fig. 1 and supplementary material Table S1).

### CPTI lines localising in the nucleus

**Fig. 2.**
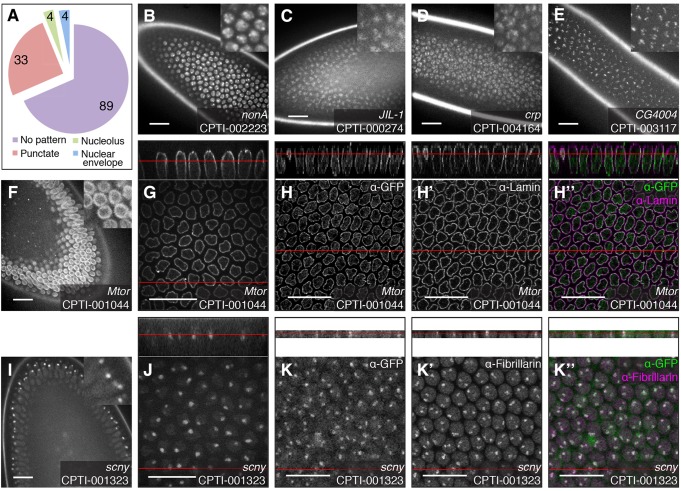
**Examples of nuclear localisation patterns of the CPTI lines at cellularisation.** (A) Distribution of nuclear patterns at stage 5. No pattern indicates a uniform distribution in the nucleoplasm. (B-E) Example of punctate patterns observed during screening in live embryos: CPTI-002223, inserted in *nonA*; CPTI-000274, inserted in JIL-1; CPTI-004164, inserted in *crp*; CPTI-003117, inserted in *GC4004*. Localisation of CPTI-001044 (inserted in *Megator*) in the nuclear membrane during screening (F) and at higher magnification (G) in live embryos. Fixed embryos stained for GFP (H), Lamin (H′) and merge showing the two stainings together (H″). The top panels show a cross-section taken through the *z*-stack. Localisation of CPTI-001323 (inserted in *scrawny*) in the nucleoli during screening (I) and at higher magnification (J) in live embryos. Fixed embryos stained for GFP (K), Fibrillarin (K′) and merge (K″). Red lines indicate the position of the face view in the *z*-section, and vice versa. Scale bars: 20 μm.

The 130 nuclear YFP-trap proteins insert in 95 genes (supplementary material Table S2 and Fig. 2). Most of these lines (89) show a uniform distribution in the nucleoplasm, the Mapmodulin insertions being amongst the brightest ones. The remaining lines have discrete localisation patterns and thus will be invaluable to analyse nuclear regionalization in live tissues (Mao et al., 2011). Thirty-three lines showed distinct punctate distributions and it is likely that many of these represent functional regions of the nucleus (Fig. 2B-E). For example, CPTI-002223 (Fig. 2B) is inserted in *no on or off transient A* (*nonA*) which encodes an hnRNP associated with omega speckles (Onorati et al., 2011). CPTI-000274 (Fig. 2C) tags JIL-1, a tandem kinase which marks active chromatin (Regnard et al., 2011). CPTI-004164 (Fig. 2D) is inserted in *cropped* (*crp*), an uncharacterised member of the basic helix-loop-helix gene superfamily (bHLHe63) (Skinner et al., 2010). Strikingly, for two lines, the puncta were localised in the apical region of the nucleus: these are CPTI-001383 and CPTI-003117, that tag respectively the genes *female sterile (1) homeotic* [*fs(1)h*] and *CG4004* (Fig. 2E). Fs(1)h is a Trithorax group protein (Strubbe et al., 2011) and the orthologue of human Brd4, while CG4004 codes for a MADF domain containing DNA binding protein of unknown function (FlyBase). Interestingly, CG4004 was found recently in a screen searching for proteins binding to TAS telomeric sequences (Antao et al., 2012). The significance of the apical localisation of these proteins in the nucleus is unknown, although this might represent discrete localisation within the nucleoli or in the perinucleolar region, since the nucleoli are apical at stage 5 (see below). In addition to the 33 punctate lines, 4 lines showed an enrichment at the nuclear periphery that resembled the nuclear envelope (Fig. 2F) and 4 lines were enriched in one or two large spots near the apical side of the nucleus, which resembled nucleoli (Fig. 2I). We confirmed localisation at these major nuclear compartments, as described below.

#### Localisation to the nuclear envelope at stage 5

The protein traps in both the nucleoporin Megator (Mtor) (Fig. 2G-H″) and the importin β1 homologue Female sterile (2) Ketel (data not shown) colocalise with the nuclear envelope marker Lamin in stage 5 embryos. To demonstrate its usefulness in live studies, we made a movie of Mtor-YFP showing the nuclear envelope breakdown and reassembly during mitosis (supplementary material Movie 1). Two other protein trap lines, CPTI-000199 and CPTI-001237, have a similar localisation (not shown) and thus we infer that these are localising at the nuclear membrane too. CPTI-000199 is inserted in the gene encoding the exportin homologue *CAS/CSE1 segregation protein.* Thus, 3 out of 4 tagged proteins found enriched at the nuclear membrane have previously been reported to be part of the nuclear transport machinery in *Drosophila* (Mason and Goldfarb, 2009). The 4th insertion, CPTI-001237, falls towards the 5′ end of the *mub* locus, although the genomic location is not in frame with the current gene model (FlyBase). *mub* is a sex-specific regulator of alternative splicing (Telonis-Scott et al., 2009). Only half of the progeny from CPTI-001237 show YFP expression suggesting that the trapped gene is sex-regulated. Thus, it is likely that CPTI-001237 tags a yet unidentified 5′ translated exon encoding a sex-specific isoform of *mub*. Its localisation at the nuclear envelope is intriguing and could be linked to a role of the inner nuclear membrane in repressing gene expression (Collas et al., 2014).

#### Localisation to the nucleolus at stage 5

The protein traps inserted in *scrawny* (CPTI-001323, Fig. 2I-K″) and in CG4038 (CPTI-002287, not shown) colocalise with the nucleolar marker Fibrillarin to one or two large puncta in the apical part of the nucleus. Scrawny is an ubiquitin-specific protease, homologue of human USP36 and yeast UBP10, which deubiquitylates histone H2B and functions in gene silencing (Buszczak et al., 2009). A strong enrichment in the nucleolus was noted during oogenesis and spermatogenesis, which we confirm here in the embryo. CG4038 is homologous to human GAR1 (and yeast Gar1p), a H/ACA box snoRNP that colocalises with Fibrillarin in human cells (Pogacic et al., 2000). Based on their similar localisation pattern, we infer that both CPTI-002443 and CPTI-002785, which tag CG11920 and CG11030 respectively, are enriched in the nucleolus. These are uncharacterised but nevertheless have features consistent with a role in nucleolar function. CG11920 has sequence homology with Imp4p, a U3 snoRNP with a box C/D motif, which is required for the early cleavage steps in pre***-***rRNA processing (Mayer et al., 2001). CG11030 harbours a Sas10/Utp3/C1D domain (FlyBase) found in Utp3 and LCP5, which are components of the U3 ribonucleoprotein complex. Thus, these localisations identify novel nucleolar factors in *Drosophila*.

### CPTI lines localising in the cytoplasm

**Fig. 3.**
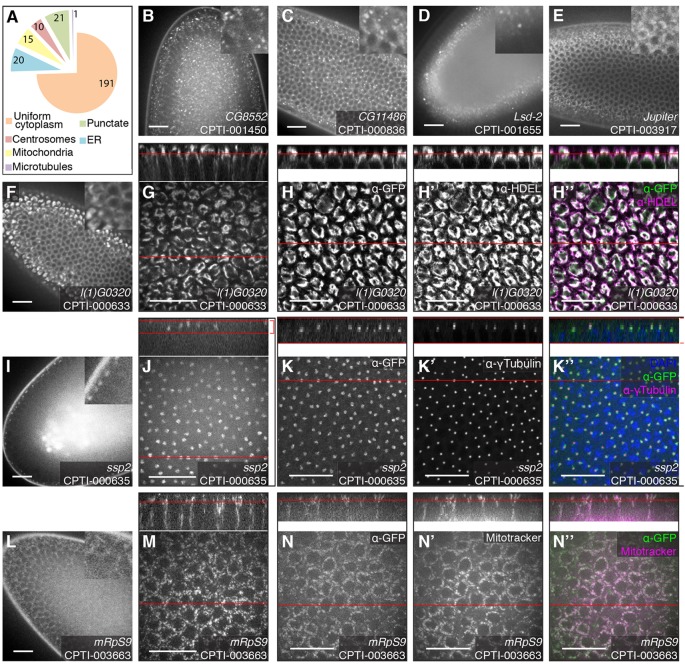
**Example of cytoplasmic localisation patterns of the CPTI lines at cellularisation.** (A) Distribution of cytoplasmic patterns at stage 5. (B-D) Examples of punctate localisation in the cytoplasm: CPTI-001450 in *CG8552* marks the Golgi apparatus and ER-to-Golgi transport vesicles (B); CPTI-000836 in *CG11486* marks P-bodies (C); and CPTI-001655 in *Lsd-2* labels the lipid droplets at the basal end of the cells. (E) CPTI-003917 inserted in *Jupiter* labels the microtubule cytoskeleton. (F-H″) The localisation of CPTI-000633 in *l(1)G0320* to the endoplasmic reticulum appears as a dense perinuclear pattern that is especially strong above the apical side of the nucleus, in live tissue during screening (F) and at high magnification (G). (H-H″) Fixed embryo stained for GFP (H) and HDEL, an ER marker (H′) and merge (H″). (I-K″) CPTI-000635 in *ssp2* localises to the centrosomes, recognizable as a pair of puncta on opposite sides of the nucleus, in the apical part of each cell. Live tissue during screening (I) and at high magnification (J). Fixed embryo stained for GFP (K) and the centrosome marker γ Tubulin (K′), and merge with DAPI in blue (K″). (L-N″) Localisation to the mitochondria of CPTI-003663, inserted in Mitochondrial ribosomal protein S9, gives the cytoplasm a granular appearance. Live tissue during screening (L) and at high magnification (M). Live embryo permeabilised and treated with Mitotracker dye to mark the mitochondria: GFP (N), Mitotracker (N′) and merge (N″). Red lines indicate the position of the face view in the *z*-section, and vice versa. Scale bars: 20 μm.

The 258 cytoplasmic YFP-trapped proteins insert in 207 unique genes (supplementary material Table S3 and Fig. 3). Most of the lines (191) show a uniform distribution in the cytoplasm, with insertions in *elf* (CPTI-00609) and *growl* (CPTI-02016) being amongst the brightest ones. The other lines showed enrichments consistent with localisation to centrosomes (10 lines), mitochondria (15 lines) and endoplasmic reticulum (20 lines) (supplementary material Table S3A and see below). One line (CPTI-003917), coding for the microtubule binding-protein Jupiter, showed a perinuclear pattern that could be distinguished from a nuclear envelope localisation (Fig. 3E), consistent with the localisation of a GFP trap in the same protein (Karpova et al., 2006). The remaining lines (21) showed punctate localisations (examples in Fig. 3B-D). Some of these are likely to represent localisation to various organelles of the secretory pathway, including the Golgi, which is dispersed in *Drosophila* (Kondylis and Rabouille, 2009) (see below).

#### Localisation to centrosomes at stage 5

An insertion in *short spindle 2* (*ssp2*) colocalises with the centrosomal marker gamma-tubulin (Fig. 3I-K″), in a pair of puncta apical to the nucleus. Ssp2, a microtubule plus end–tracking protein involved in spindle assembly, had not been previously reported to localise to centrosomes (Goshima et al., 2007; Li et al., 2011) and was not found in a *Drosophila* S2 cell RNAi screen to find factors required for centrosome function (Dobbelaere et al., 2008). The centrosomal localisation could be specific to early embryos, as it disappears after stage 9 (data not shown). In total, 10 CPTI lines localised to centrosomes at stage 5, tagging 5 genes including *ssp2*. Three viable insertions in 14-3-3epsilon and two insertions in calmodulin (one lethal and one viable) are found enriched in centrosomes. The latter is consistent with GFP-calmodulin localisation to centrosomes in S2 cells (Dobbelaere et al., 2008). CPTI-03513 falls in the *pathetic* (*path*) locus and is found both in the cytosol and in a centrosome-like enrichment. The insertion is 5′ to the ATG so either the current gene model is incorrect or Path is not the protein tagged. Path codes for a Proton-assisted Amino acid Transporter (PAT) whose localisation has been examined using a GFP fusion in S2 cells and larval fat bodies: a lysosome-like enrichment rather than a centrosome-like one was reported (Ogmundsdottir et al., 2012). Finally, three viable insertions in the *gsk-3* homologue exhibit a centrosomal localisation in embryos, consistent with similar observations with a GFP-protein trap in embryos (Bobinnec et al., 2006).

#### Localisation to the mitochondria at stage 5

CPTI-003663, an insertion in the Mitochondrial Ribosomal Protein S9 (mRpS9), shows a granular signal in the cytoplasm (Fig. 3L). At higher magnification the signal is resolved into discrete speckles (Fig. 3M) that colocalise with the marker Mitotracker, showing that these are mitochondria (Fig. 3N-N″). The same localisation is found for CPTI-000877, tagging another mitochondrial protein, the beta subunit of the F0/F1 ATP synthase. In total, 15 lines showed a mitochondria-like localisation, corresponding to 11 genes (supplementary material Table S3). Several insertions fall into known genes, some of which have been previously linked with mitochondrial function. A single insertion in Dacapo, the homologue of p27, a cyclin-dependent protein serine/threonine kinase inhibitor, localises both in the nucleus and in a mitochondria-like pattern (supplementary material Tables S2 and S3). Interestingly, Dacapo is activated by mitochondria dysfunction (Owusu-Ansah et al., 2008). Three insertions in Larp, containing a 1 HTH La-type RNA-binding domain, show a mitochondria-like localisation at stage 5 and are expressed in somatic muscles later in embryogenesis. Larp is known to localise to mitochondria in early spermatocytes, where it might be required for mitochondrion inheritance (Ichihara et al., 2007). Mdh2, homologous to mitochondrial malate dehydrogenase, is tagged by one insertion and was shown to localise to mitochondria in salivary glands (Wang et al., 2010). Another line, CPTI-001595, inserts into the *split ends* (*spen*) gene, coding for a RNP protein found in nuclei (Wiellette et al., 1999). Since CPTI-001595 localises to mitochondria but not the nuclei, this is inconsistent with this insertion tagging Spen. The insertion sequence of CPTI-001595 maps to the 5′UTR of *spen*, upstream of the ATG, so it could trap another gene at this location (or the sequenced insertion is not responsible for the observed YFP localisation in mitochondria). The remaining lines tag 5 uncharacterised genes: CG10602, CG1640, CG18769, CG3902 and CG7985. CG18769, tagged by 2 lines, is homologous to the mitochondrial calcium uniporter, while CG3902, a member of the acyl-CoA dehydrogenase family, was identified as a mitochondrial protein by mass spectrometry analysis (Alonso et al., 2005). These localisations are thus likely to identify new mitochondrial factors.

#### Localisation in the ER at stage 5

In embryos, the endoplasmic reticulum is enriched apically to the nucleus as shown by the localisation of CPTI-000633, tagging *lethal(1)G0320*, the orthologue of SSR1, a component of the ER translocon (Fig. 3F-H″ and supplementary material Movie 2). In immunostainings, l(1)G0320-YFP colocalises with the epitope HDEL, a marker of ER resident proteins (Fig. 3H-H″). Using this line, supplementary material Movie 2 illustrates how the ER maintains its integrity during cell division, in contrast to the nuclear envelope (supplementary material Movie 1). CPTI-000633 shows some tissue specificity and is expressed more strongly in the epidermis, the salivary glands and the tracheal system in late embryogenesis (supplementary material Table S1). In total, 20 lines inserted in 18 genes exhibit an ER-like apical enrichment at cellularisation. The difficulty with ER localisation is that it could arise if misfolding YFP-trap proteins are retained through the ER. The localisation is likely to be genuine for 5 insertions into 4 genes that are either known or predicted to code for ER resident proteins: two components of the ER translocon: l(1)G0320 (SSR1, already mentioned above) and Sec61alpha; Protein Disulfide Isomerase (PDI), required for protein folding within the ER lumen (Ni and Lee, 2007) and Reticulon-like 1 (Rtnl1), a marker of smooth ER tubules (Roper, 2007). For the other proteins (supplementary material Table S3), the ER localisation might or might not be correct. Some of the lines are homozygous lethal which might indicate a defect with the protein-trap, and complementation tests will be required to test if the lethality is associated with the gene tagged by the YFP exon.

#### Cytoplasmic punctate localisation at stage 5

Twenty-one lines inserted in 15 genes localise in puncta (supplementary material Table S3). For some lines the puncta are enriched on the basal side of the cell, as exemplified by insertions in *lsd-2*, a marker of lipid droplets (Fig. 3D). We expected some punctate lines to label vesicles of the secretory pathway such as endosomes or lysosomes, but also the Golgi (Sisson et al., 2000). Indeed, CPTI-004256 and CPTI-004394 tag *rab1*, a small GTPase involved in ER-Golgi trafficking (Kondylis and Rabouille, 2009). CPTI-001718, with basally enriched puncta, tags Visgun, the *Drosophila* orthologue of Endolyn, a sialomucin which localises to endolysosomal organelles both in *Drosophila* and human cells (Zhou et al., 2006). CPTI-001775 tags the *Drosophila* homologue of the lysosome-associated membrane protein Lamp1 (Pulipparacharuvil et al., 2005). CPTI-000038 and CPTI-002401 tag *Fe1HCH* and CPTI-100064 tags *Fe2LCH*, the two genes encoding the ferritin complex in *Drosophila*. All 3 lines localise in basal puncta, consistent with the trafficking of iron-loaded ferritin through the Golgi (Missirlis et al., 2007). However, these lines are lethal and show also an ER localisation, which might indicate a defect in trafficking. CPTI-001962 and CPTI-003653 tag *short stop* (*shot*), the *Drosophila* homologue of spectraplakins. One line is lethal and show large puncta which could be non-specific aggregates, while the other is viable. Shot binds microtubules and localises to the fusome (Roper and Brown, 2004), so the puncta in the viable line could represent some association of Shot with vesicles of the secretory pathway.

The remaining punctate lines label organelles or regions distinct from the secretory pathway. CPTI-002786 inserts in the *Drosophila* homologue of catalase, the sole enzyme in insects known to eliminate hydrogen peroxide (Orr and Sohal, 1992) and is thus likely to label the peroxisomes. Other lines tag ribonucleoproteins involved in mRNA transport and regulation: Imp (CPTI-004117) is a RNA-binding protein and Me31B (CPTI-003927) a RNA helicase found in RNP granules (McDermott et al., 2012), while Pumilio (CPTI- 002853) represses the translation of specific mRNAs such as *hunchback*. All CPTI lines tagging these genes are later enriched in the CNS in late embryogenesis (supplementary material Table S1). Other lines tag two kinases CaMKII (CPTI-000944) and Drk (CPTI-002249, note that the main localisation is in nuclei).

We also found two unknown genes tagged by several lines each, that exhibit a striking punctate localisation at cellularisation: CG8552 (4 lines, including CPTI-001450, Fig. 3B) and CG11486 (2 lines, including CPTI-000836, Fig. 3C). Sequence homologies suggest roles in trafficking and RNA regulation, respectively: CG8552 is an orthologue of SEC23IP involved in ER-to-Golgi trafficking and CG11486 is an orthologue of PAN3, a regulatory component of the deadenylation complex that binds to poly(A)-binding protein.

### CPTI lines localising at the cortex, membrane or extracellular space

**Fig. 4.**
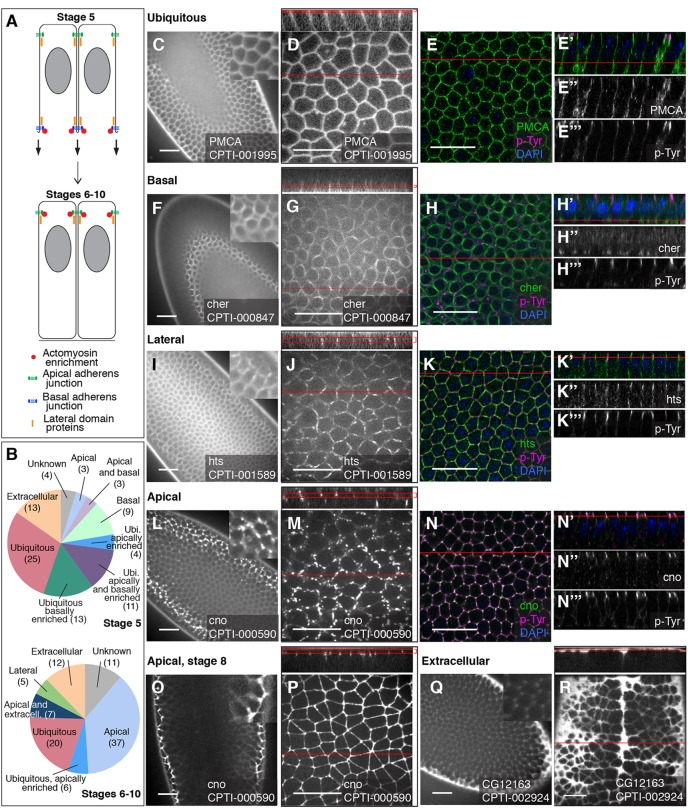
**Examples of apico-basal localisations of membranous/cortical and extracellular YFP-tagged proteins.** (A) The position of adherens junctions, lateral proteins and actomyosin cytoskeleton at stage 5 (cellularisation) and stages 6 to 10 (early morphogenesis). At stage 5, a transient basal junction forms. (B) Distribution of the lines along the apico-basal domains of the plasma membrane and presence in the extracellular space, at stage 5 and stages 6 to 10. (C-E‴) Example of ubiquitous membranous localisation: CPTI-001995, inserted in the plasma membrane calcium ATPase (PMCA). Live embryos at stage 5 during screening (C) and at high magnification (D). Co-staining of fixed stage 5 embryos with anti-GFP and the adherens junction marker p-Tyr, with nuclei labelled by DAPI in blue: merge face view (E) and *z* section (E′); GFP staining (E″) and p-Tyr staining (E‴). PMCA-YFP is distributed throughout the plasma membrane. (F-H‴) Example of basal localisation at stage 5, at the cellularisation front: CPTI-000847, inserted in Cheerio. Live embryos at stage 5 during screening (F) and at high magnification (G). Co-staining of fixed stage 5 embryos: merge GFP and p-Tyr stainings for face view (H) and *z* section (H′); GFP only (H″) and p-Tyr only (H‴). In face views, Cheerio-YFP localises in a ring-like pattern typical of the actomyosin-rich cellularisation front. (I-K‴) Example of lateral localisation at stage 5: CPTI-001589, inserted in *hu li tai shao* (*hts*). Live embryos at stage 5 during screening (I) and at high magnification (J). Co-staining of fixed stage 5 embryos: merge GFP and p-Tyr stainings for face view (K) and *z* section (K′); GFP only (K″) and p-Tyr only (K‴). In *z* sections, Hts-YFP localises just below and above the apical and basal junctions at stage 5, which is typical of the localisation of lateral proteins at cellularisation (see A). (L-N‴) Example of apical localisation at stage 5: CPTI-000590, inserted in *canoe* (*cno*). Live embryos at stage 5 during screening (L) and at high magnification (M). Co-staining of fixed stage 5 embryos: merge GFP and p-Tyr stainings for face view (N) and *z* section (N′); GFP only (N″) and p-Tyr only (N‴). At stage 5, most but not all the Cno-YFP signal has reached an apical position and colocalises with p-Tyr. Low (O) and high (P) magnification in live embryos at stage 8: all Cno-YFP is now apical and forms a continuous belt at the apical junctional domain. (Q,R) Example of extracellular localisation: CPTI-002924, inserted in GC12163. Live embryos during screening at stage 5 (Q) and stage 8 (R). The YFP signal pools in gaps between the apical ends of the cells (Q,R) and in the groove formed by the ventral midline (top to bottom groove in R). In all images, red lines indicate the position of the *z* section in the face view, and vice versa. When face views are projections of several *z* planes, two red lines in the *z* section indicate the bottom and top-most planes used for the projection. Scale bars: 20 μm.

For the lines localising at the membrane/cortex or the extracellular space, we broadened our characterisation from stage 5 throughout to stage 10, to cover epithelial morphogenesis, increasing the number of lines examined to 102 (supplementary material Table S4 and Fig. 4), as described in the following sections.

#### Apico-basal localisations at stage 5 to 10

We identified the apico-basal localisation of representative lines in fixed tissues by comparing it with the localisation of a phosphotyrosine epitope (p-Tyr), which is enriched at adherens junctions (Fig. 4) (Muller and Wieschaus, 1996). From this, we inferred the position along the apico-basal axis of all membranous/cortical lines with comparable localisations (supplementary material Table S4). The epithelium of stage 5-10 embryos is still polarizing and the junctional and membranous domains are immature compared to later epithelia (Tepass and Hartenstein, 1994; Huang et al., 2011). Ultrastructural studies showed that the zonula adherens becomes mature around stage 11, consisting before that of spot adherens junctions progressively coalescing (Tepass and Hartenstein, 1994). The septate junctions (functionally analogous to vertebrate tight junctions) have not formed yet. There is no basement membrane either, however an apical lamina is seen from stage 10 onwards, suggesting that an apical extracellular matrix is being deposited well before a basal one (Tepass and Hartenstein, 1994). The localisations we find throughout stage 5 to 10 are consistent with these earlier findings, as detailed below.

##### Ubiquitous localisations

Many protein traps were detected ubiquitously at the membrane at stage 5 before resolving into either apical (mainly) or lateral membrane localisations at stages 6 to 10 (Fig. 4A,B and supplementary material Table S4). Some proteins keep their ubiquitous localisation at the membrane throughout, such as the plasma membrane calcium ATPase (PMCA) (Fig. 4C-E‴). Bright lines include CPTI-004113, inserted in Gilgamesh, shown in supplementary material Movie 3 labelling cell membranes during axis extension. In total, 53 lines are found ubiquitously at the membrane at stage 5, and half of these are also enriched apically, basally or both (Fig. 4B and supplementary material Table S4). This number decreases to 26 lines at stages 6 to 10, with 6 apically enriched (Fig. 4B and supplementary material Table S4).

##### Basal localisations

At stage 5, basal localisation or enrichment can represent either enrichment at the cytokinetic apparatus at the cellularisation front or at the so-called basal adherens junctions that form transiently during cellularisation (Hunter and Wieschaus, 2000; Mazumdar and Mazumdar, 2002). Thirty-six lines (inserted in 29 genes) showed some basal enrichment at stage 5. A group of insertions appear to localise principally at the cellularisation front: these include *Abelson kinase*, *amphiphysin*, *cheerio* (the homologue of filamin) (Fig. 4F-H‴), *cindr* (the CIN85/CD2AP orthologue), the Ste20 kinase *mishappen*, the unconventional *Myosin 31DF* and *zipper* (coding for Myosin II heavy chain). Insertions in these proteins relocalise apically at stages 6 to 10 (except for Amphyphisin-YFP and Cindr-YFP which become cytoplasmic). From this list, proteins known to be at the cellularisation front are Myosin II (Young et al., 1991), and Amphiphysin, a BAR domain protein (Sokac and Wieschaus, 2008; Su et al., 2013). A larger group of insertions are found both apically and basally enriched at stage 5. For some of these, the basal enrichment looks like an enrichment at the cellularisation front, but for others the enrichment resembles more an enrichment at the transient basal adherens junctions (data not shown). These include insertions in *alpha-catenin* and in *armadillo* (the beta-catenin homologue). The large majority of these insertions became apically localised at stages 6 to 10 (supplementary material Table S4). We did not find any proteins localising basally at stages 6 to 10, which is consistent with the absence of known basal membrane and extracellular matrix markers at these early stages.

##### Lateral localisations

Although this distinction is difficult to make at stage 5, lateral localisation was distinguishable from an apical/junctional localisation for a few insertions at stages 6 to 10 (supplementary material Table S4): two insertions in *disc-large* (*dlg*), and single insertions in *hu li tai shao* (*hts*) (stage 5 shown in Fig. 4I-K‴), *tropomodulin* and an uncharacterised gene, *CG42748*. From those, Dlg is a known marker of the lateral membrane, and the lateral localisation of Hts was reported recently (Wang et al., 2011).

##### Apical localisations

Forty-four insertions were enriched apically at stages 6-10 (supplementary material Table S4). This includes localisations to the junctional domain, but also localisations which might be more apical (St Johnston and Sanson, 2011). Lines in genes known to localise to the apical junctional domain include insertions in *canoe* (the afadin homologue) and *echinoid* (the nectin homologue) (Harris, 2012). At stage 5, Canoe-YFP is localised in a spot-like manner at the membrane, with some spots having not yet reached an apical position (Fig. 4L,M). Canoe-YFP colocalises with p-Tyr in immunostainings (Fig. 4N-N‴). At stage 8 the apical localisation matures into a continuous cortical signal (Fig. 4O,P). Canoe exemplifies how apical junctional localisations mature through early development (Sawyer et al., 2009). Other lines are already apical at stage 5 including an insertion in *arpc2*, encoding a component of the Arp2/3 complex, and an insertion in *CG6398*, an uncharacterised gene with homology to Claudins (Wu et al., 2004) (supplementary material Table S4). This is in contrast with other Claudin homologues in *Drosophila* which localise laterally or later at septate junctions (Hall et al., 2014). CG6398-YFP is a notably bright line and later in embryogenesis labels strongly all apical surfaces including those of tubular secretory epithelia (trachea, salivary glands, hindgut; see supplementary material Table S1). The insertions in *arpc2* and *CG6398* are also striking for their apical cap-like (medial) enrichment: we noted similar enrichment in addition to a cortical apical signal for about half of the apical lines at stages 6-10 (supplementary material Table S4).

##### Extracellular localisations

Thirteen lines (inserted in 9 genes) are detected in the extracellular space at stage 5 (supplementary material Table S4). The YFP tagged-proteins fill all the space available between the apical surface of the cells and the vitelline membrane. At stages 6 to 10, they accumulate strongly above dividing cells and the ventral midline groove because these are slightly deeper than the rest of the epidermis. This is illustrated by the localisation of CPTI-002924 at stage 5 and 8 (Fig. 4Q,R; see also supplementary material Fig. S1C), one of two insertions in CG12163, a likely homologue of Cathepsin F (Kocks et al., 2003). Cathepsins are cysteine proteases which have been found to be associated with endosomes, lysosomes and the extracellular space (Brix et al., 2008). In the case of CG12163-YFP, we find a very clear extracellular localisation but no cytoplasmic localisation (supplementary material Table S1). Two other proteases, Kuzbanian and Neu3/Meltrin, are ADAM metalloproteases of the M12B family (Meyer et al., 2011). Both genes are tagged by 2 insertions, but for Kuzbanian only one is extracellular (supplementary material Table S1). The other insertion has an ER localisation which could indicate a secretory defect (supplementary material Table S3). Quasimodo/CG13432 codes for a Zona Pellucida domain-containing protein expressed in the epidermis and is tagged by a single insertion (supplementary material Table S1). Consistent with this, ZP-domains proteins are known to be cleaved to release an extracellular domain and are components of the apical ECM in epidermal and neuronal cells (Plaza et al., 2010). Interestingly, Quasimodo was recently found to act in the clock neuronal circuit (Chen et al., 2011). Another insertion tags *dsx-c73a*, and our data corroborate an earlier study with an antibody showing that the protein is secreted (Andrew and Baker, 2008) (supplementary material Tables S1 and S4). This work and ours show that Dsx-c73a is more strongly expressed in the epidermal cells that will form trichomes, and also in internal tissues such as the tracheal system and pharynx, suggesting a role as an apical ECM protein (supplementary material Table S1). The other insertions with extracellular localisation include Frazzled, a transmembrane protein; Lamp1 (but the main localisation is in cytoplasm, see previous section) and Dally-like, a glypican homologue (the latter might represent a defective localisation as the insertion is lethal and also localises to the ER) (supplementary material Table S4). Finally, three insertions tag the extracellular domain of Sidekick, a transmembrane protein of the immunoglobulin family, and are notably enriched at tricellular vertices (supplementary material Table S4 and see below). In addition to these localisations at stage 5 to 10, we found a few insertions whose expression is not detectable at stage 5, but are clearly extracellular at stage 11 and/or stage 16 (supplementary material Table S1). These insertions are in *babos*, *CG10992* (homologue of Cathepsin B; see Kocks et al., 2003), *CG32066*, *CG8213* (a serine-type endopeptidase), *chitin deacetylase-like 4*, *dally* (Glypican homologue) and *Fasciclin 2*.

#### Localisation of cortical/membranous proteins at tricellular vertices

**Fig. 5.**
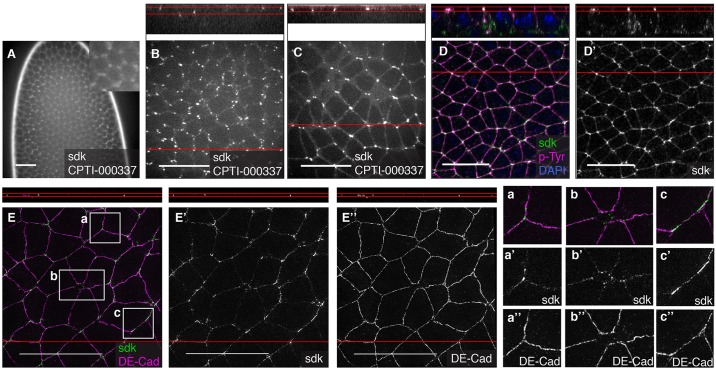
**Localisation of Sidekick at tricellular vertices.** (A,B) In live embryos at stage 5, CPTI-000337 inserted in Sidekick localises in spots that become progressively enriched apically, at cell vertices. (C) In live embryos at stage 8, Sidekick-YFP is localised apically and marks cell vertices between three or more cells. (D,D′) In fixed embryos of same stage, co-staining with p-Tyr confirms the apical junctional position of Sidekick-YFP. (E-E″) Superresolution imaging of fixed embryos at stage 8 stained for DE-Cadherin and YFP shows that DE-Cadherin and Sidekick tend to localise in complementary domains, with DE-Cadherin mainly at bicellular contacts (E,E″,a″,b″) and Sidekick (E,E′) at tricellular (a′) or multicellular contacts (b′). Occasionally, Sidekick forms plaques at bicellular contacts where Cadherin is less enriched (c′,c″). Top panels show side views from the reconstruction of the *z* planes at the position of the red line in the main images. Scale bars: 20 μm.

A subset of proteins were found enriched at tricellular vertices where the corners of three (or more) cells meet. The most striking of these are three insertions in Sidekick (Sdk), a transmembrane protein with a large extracellular region containing immunoglobulin and fibronectin domains (Nguyen et al., 1997) (Fig. 5). Sdk-YFP initially localises as spots at stage 5 that appear more concentrated in the vicinity of tricellular vertices and have not all reached an apical position (Fig. 5A,B). At stage 8 and beyond, Sdk-YFP becomes fully apical and is also mostly excluded from bicellular contacts (Fig. 5C). This is confirmed by a co-staining with p-Tyr (Fig. 5D,D′). Although Sidekick is the only protein found in the screen whose membranous localisation is mostly at vertices, we found a large number of membranous/cortical proteins that are *enriched* there at stages 6 to 10 (30 lines inserted in 24 genes, supplementary material Table S4). The majority are apically localised and include Canoe, whose enrichment at vertices was previously reported in early embryos (Sawyer et al., 2009). Consistent with the vertex enrichment of actin also reported in that study, most proteins localising at tricellular vertices in our screen are actin-binding proteins or actomyosin regulators (supplementary material Table S4).

Later during embryogenesis, tricellular vertices develop specialized junctions, the tricellular junctions, which maintain the permeability barrier at the corners of the cells in mature epithelia. Tricellular junctions are part of the septate junctions in insects and of the tight junctions in vertebrates. In *Drosophila*, the only protein exclusively found in tricellular junctions is Gliotactin, a member of the Neuroligin family (Padash-Barmchi et al., 2013). Gliotactin is not expressed in early embryos, is detected faintly at stage 11, and only found associated with tricellular vertices from stage 13 onwards (Schulte et al., 2003). Consistent with this, the sole CPTI insertion in Gliotactin does not show detectable expression at stage 5 or 11, but is robustly expressed later in embryogenesis (supplementary material Table S1) and localises to tricellular junctions [see companion paper (Lowe et al., 2014)]. Thus, Sidekick represents the second example in *Drosophila* of a protein specifically localised at tricellular vertices. By contrast to Gliotactin, this localisation occurs from the start of epithelium formation in early embryos, and it is at the level of the adherens junctions, not the septate junctions. To examine the relative distribution of E-Cadherin, the main component of adherens junctions, and Sidekick, we imaged co-stainings of Sdk-YFP and E-Cadherin at stage 8 with superresolution microscopy (Fig. 5E-E″). Sidekick and E-Cadherin showed mostly non-overlapping localisations, although they are in the same position along the apico-basal axis. Together, our findings suggest that Sidekick marks a specialized apical junctional membrane domain at tricellular vertices.

#### Distinct localisations of cortical/membranous proteins during cytokinesis

**Fig. 6.**
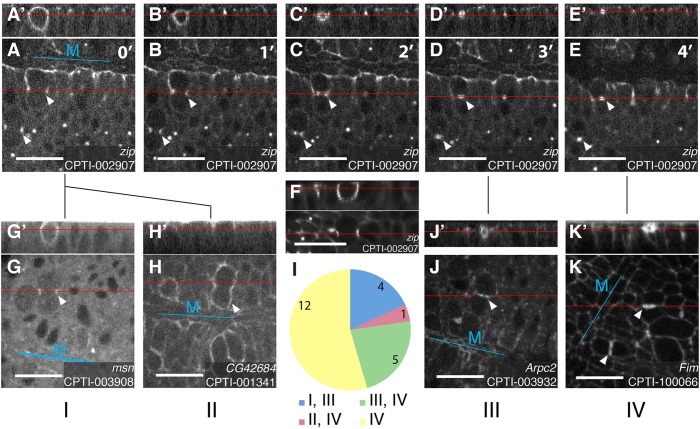
**Localisation patterns during cytokinesis.** (A-E) The localisation of CPTI-002907, an insertion in the gene *zipper* coding for Myosin II Heavy Chain, reveals the closure of the cytokinesis ring, from its formation (A) to its closure with the formation of the midbody (D,E). Side views from the reconstruction of the *z* planes show the asymmetric apico-basal shape of the cytokinesis ring (A′) and its closure from basal to apical (B′-E′). (F) An image taken by light sheet imaging, confirming the asymmetric shape of the cytokinesis ring. Membranous/cortical protein localisations at cytokinesis were classified as follows. Categories I and II correspond to the beginning of cytokinesis, I for localisation at the ring (G,G′) and II for localisation at the apical cortex (H,H′). Category III corresponds to proteins localising immediately adjacent to the midbody (J,J′) and category IV proteins localise a little later at the new membrane between daughter cells, on either side of the midbody (K,K′). The pie chart in I shows the number of insertions in each category. Cytokinesis was imaged in stage 9 or 10 embryos. Top panels show side views from the reconstruction of the *z* planes at the position of the red line in the main images. M, ventral midline. Scale bars: 20 μm.

When examining the subcellular localisation of cortical/membranous proteins at stages 6-10, we noticed some distinct patterns of localisation in dividing cells during cytokinesis (Fig. 6). The changing shape of the cytokinesis ring is illustrated by the localisation of CPTI-002907, one of the two insertions in *zipper* (Myosin II Heavy Chain). Zipper-YFP is associated with the cytokinesis ring from its formation until its closure (Fig. 6A-E). In side views, Zipper-YFP highlights the asymmetric shape of the cytokinesis ring which is wide apically and narrow basally (Fig. 6A′,F). As reported recently for several *Drosophila* epithelial tissues, the cytokinesis ring closes in a basal to apical direction and the midbody is positioned apically (Fig. 6A′-E′) (Founounou et al., 2013; Guillot and Lecuit, 2013; Herszterg et al., 2013a; Morais-de-Sa and Sunkel, 2013a).

We classified the localisations during cytokinesis into 4 categories (Fig. 6 and supplementary material Table S4). Category I corresponds to four CPTI lines that associated with the cytokinesis ring when it is wide open (Fig. 6G,G′): two insertions in *zipper* and single insertions in *misshapen* (*msn*, encoding a Ste20 kinase) and *septin 1.* In contrast to Myosin II Heavy Chain and Septin 1, a function of *msn* in cytokinesis has not been reported yet in *Drosophila*, but its homologue MINK1 is required for the abscission of mammalian cells in culture (Hyodo et al., 2012). Category II is defined by a single insertion in CG42684, an uncharacterised gene with a rasGAP domain. This line has a striking localisation at the apical cortex of dividing cells and is the only line we found with this localisation (Fig. 6H,H′). Category III corresponds to 9 lines that localise when the cytokinesis ring is closing (Fig. 6J,J′). These include Category I proteins Zipper, Msn and Septin 1 and 5 other lines that start to be localised at this late closure stage: Abelson tyrosine kinase, Arpc2 (component of the Arp2/3 complex), Cheerio (homologue of Filamin), Fimbrin and Spinophilin (supplementary material Table S4). Because all proteins in Category III are F-actin binding proteins, this suggests that these localisations correspond to the time when actin is polymerizing on either sides of the midbody (Herszterg et al., 2013b; Morais-de-Sa and Sunkel, 2013b). Corroborating the localisation of Arpc2, another subunit of the Arp2/3 complex (Arc-1) was shown to transiently localise on either side of the midbody (Morais-de-Sa and Sunkel, 2013a). Category IV corresponds to lines that localise to the new junction forming between the two daughter cells. This localisation is disc-like with an interruption in the middle, suggesting that category IV proteins are excluded from the midbody. Category III proteins Abelson tyrosine kinase, Arpc2, Cheerio, Fimbrin and Spinophilin also belong to Category IV (Fig. 6I and supplementary material Table S4). In addition, 13 more lines only belong to category IV, including insertions in 6 proteins ubiquitously localising at the membrane such as B4 (or Susi), Basigin, CG3036, CG42389, Nervana-1 and Vimar and insertions in 3 apically localised proteins: ced-12 (or ELMO), CG42684 (also in category II, see above), and Dystrophin (supplementary material Table S4). This is consistent with category IV representing localisation to the new membrane expanding between the daughter cells, which follows actin polymerization around the midbody (Herszterg et al., 2013b).

## DISCUSSION

Our systematic characterisation of the CPTI lines in embryos gives clues to the function of many uncharacterised proteins and identifies markers of organelles and subcellular regions of the nucleus (nucleoli, nuclear envelope, nuclear speckles), the cytoplasm (centrosomes, mitochondria, endoplasmic reticulum, Golgi, lysosomes and peroxisomes) and the plasma membrane or cortex (apico-basal locations, extracellular proteins, tricellular vertices, cytokinetic structures). Since the tagged proteins are expressed at endogenous levels, the usefulness of the lines will increase with the development of more sensitive imaging methods, such as light sheet (Keller et al., 2008) (Fig. 6F) or superresolution microscopy (Gustafsson et al., 2008) (Fig. 5E-E″).

The YFP-trap lines are incredibly versatile tools: they can be used to follow the dynamics of tagged proteins in real time (supplementary material Movies 1-3) and are amenable for FRAP, FLIP or CALI studies (Monier et al., 2010); the presence of P-element sequences allows one to swap the YFP exon for other exons (Gloor et al., 1991), such as encoding a different colour FP or a Gal4, and to generate mutants by imprecise excision (Voelker et al., 1984); both tagged RNA and protein can be knocked down by targeting the YFP sequence (Caussinus et al., 2012; Neumuller et al., 2012); and partners of the tagged protein can be found using mass spectrometry after endogenous complex purification with the FLAG and STREPII tags present in the YFP exon (Rees et al., 2011; Lowe et al., 2014).

## MATERIALS AND METHODS

### *Drosophila* strains

The CPTI lines were provided by the Cambridge Protein Trap Consortium (Lowe et al., 2014). The lines are insertions of a Venus YFP-bearing PiggyBac element, with the Venus YFP exon carrying StrepII and FLAG tags and flanked by splice donor and acceptor sites. Most of the constructs also contain nested P-element ends (see supplementary Methods and Fig. S1A in Lowe et al., 2014).

Some of the lines generated by the Consortium were duplicates (insertions in the same gene), and not all duplicates were made available to the screening groups, bringing the total number of CPTI lines characterised in this paper to 553. The majority of these lines are available from the Kyoto Stock Center, including some of the duplicates not characterised here. We also characterised seven pilot lines that were not given CPTI numbers, retaining their original ‘NPSV’ and ‘PPSV’ designations (gifts from N. Lowe and D. St Johnston, University of Cambridge, UK). The NPSV lines do not have nested P-elements ends.

The stocks were balanced over FM7h (first chromosome insertions), SM6a (second chromosome insertions) and TM6C(Sb) (third chromosome insertions). Fourth chromosome insertions are over *ey*^D^. We recorded whether the balancer was still present in all, some or none of the flies: this is shown in supplementary material Tables S1-S4 as the insertion being lethal, viable (floating balancer) or viable, respectively.

### Embryo collection, staining and imaging

Fly stocks were raised on standard maize meal medium. Flies were transferred to cages at 25°C and laid eggs on grape juice agar plates with yeast. Embryos were mainly imaged live, detecting YFP fluorescence. Immunostainings of fixed embryos were also performed for example lines. Details of embryo fixation and immunostaining, live imaging and superresolution imaging, and MitoTracker staining of embryonic mitochondria are provided in the methods in the supplementary material.

### Curation of the data

Imaging data from each of the 560 lines were inspected and the subcellular localisation determined. If necessary, imaging was repeated to confirm the localisation. Example lines were selected to illustrate the patterns found and to confirm the localisation to main subcellular compartments by immunostaining in fixed tissues (Figs 2-6). The subcellular localisation data were curated and presented in four sortable Excel tables (supplementary material Tables S1-S4). The expression patterns were also recorded when obvious, but were not characterised as exhaustively. The primary characterisation of all the lines in this paper was carried out without knowing the identity of the genes tagged, as gene identity was released by the Consortium post-annotation in Flyprot. Gene names, symbols and FlyBase IDs are concordant with FlyBase, version FB2013_06, released November 4, 2013 [see flybase.org and St Pierre et al. (2014)].

The tables also contain information taken from Flyprot: gene linked to each insertion (388 genes in total), chromosome location and insertion sequence. For more information on these elements, see the stock reports in www.flyprot.org and also Lowe et al. (2014). We have also evaluated the evidence in support of a given line being a protein trap, based on the in-frame information and further notes and evidence listed in Table S2 in Lowe et al. (2014), as well as the localisation data in this paper. When the evidence was strong enough, we have listed a ‘yes’ in this column (477 lines). When the evidence was missing or ambiguous, we have listed a ‘no’.

## Supplementary Material

Supplementary Material
