# Supplementary material for: Subcellular localisations of the CPTI collection of YFP-tagged proteins in Drosophila embryos
Source: Development. 2014 Oct;141(20):4006–17. doi: 10.1242/dev.111310 (PMC4197698; doi:10.1242/dev.111310)
Supplement: Supplementary Material [file supp_141_20_4006__index.html]

Supplementary Material 

# Subcellular localisations of the CPTI collection of YFP-tagged proteins in *Drosophila* embryos

## DEV111310 Supplementary Material

**Files in this Data Supplement:**

- **Supplementary Material**
