# Supplementary material for: Subcellular localisations of the CPTI collection of YFP-tagged proteins in Drosophila embryos
Source: Development. 2014 Oct;141(20):4006–17. doi: 10.1242/dev.111310 (PMC4197698; doi:10.1242/dev.111310)
Supplement: Supplementary Material [file supp_141.20.4006_DEV111310.pdf]

Imaging Methods

Imaging of live embryos

Embryos were dechorionated by immersion in 50% sodium hypochlorite solution for 2 minutes, mounted on slides in a shallow well filled with halocarbon oil (Voltalef PCTFE, Arkema) and covered with a coverslip. They were imaged on a Nikon E1000 microscope with a Yokogawa CSU10 spinning disc confocal scanner unit, and illuminated using a Spectral Applied Research LMM5 laser module (491 nm for YFP excitation; 561 nm for Mitotracker Red). Images were captured using a Hamamatsu EM-CCD camera and Volocity software. Embryos were staged according to Wieschaus and Nusslein-Volhard (1998).

Low-magnification screen of all CPTI lines: Batches of embryos encompassing stages 5-17 were mounted together. Stage 11 and late (stage 15-17) embryos were imaged using a 20× NA=0.75 air objective lens (giving only semi-confocal resolution). Stage 5 embryos were imaged using a 40× NA= 1.3 oil-immersion objective lens. Single *z*-planes were selected and imaged individually, with exposure settings varied in accordance with the fluorescence intensity of the line.

Higher magnification screen of plasma membrane/cortex-localised CPTI lines: Embryos were mounted individually and confocal stacks (images separated by 0.5 μm in z) of the ventral side of stage 6, 7, 8, 9 and 10 embryos were imaged using a 60× NA=1.4 oil-immersion objective. We took stacks of a total depth of 35 μm at stage 6 and 7 and 25 μm at stage 8, 9 and 10. At the end of cellularisation (stage 5), ventral cells are approximately 40 μm tall, but shorten to approximately 25-30 μm after mesoderm invagination (which is complete by stage 7) with cells shortening further during germband extension (stage 7-10). Thus, at each stage we imaged the full apical-basal lengths of the ventral-most cells of the embryo (imaging in the anterior part of the trunk).

High magnification live imaging of example nuclear and cytoplasmic lines (Figs 2 and 3): Stage 5 embryos were mounted individually and confocal stacks (images separated by 0.5 μm in z) of the ventral side were acquired using a 100× NA=1.4 oil-immersion objective.

Light sheet imaging: A digitally scanned light sheet microscope (DSLM) (Keller et al., 2008) was used in Fig. 6H to confirm the asymmetric shape of the actomyosin cytokinetic ring in live embryos that had been observed by spinning disc confocal microscopy. Imaging was performed using a 488 nm laser on a custom-made microscope with a 25× NA=1.1 water-dipping detection Nikon objective, 10× NA=0.3 water dipping excitation Nikon objective and Hamamatsu Orca Flash 4.0 V2 camera. The light sheet thickness used was 2 μm (Laser Analytics Group and Cambridge Advanced Imaging Centre, University of Cambridge).

Movies: Stage 8 embryos were individually mounted in halocarbon oil in a well between two coverslips on an oxygen-permeable membrane (Lumox, IVSS), and covered with a coverslip. Confocal stacks with 1 μm *z*-spacing of the ventral side were acquired every minute, with a 100× NA=1.4 oil-immersion objective for the nuclear envelope and endoplasmic reticulum movies (Movies 1 and 2), and a 60× NA=1.4 oil-immersion objective for the membrane movie (Movie 3). The membrane movie is displayed as a maximum-intensity projection of three *z*-planes; the nuclear envelope movie as a maximum-intensity projection of two *z*-planes; and the endoplasmic reticulum movie as a single *z*-plane. Movement in *z* of the embryos was corrected by selecting appropriate *z*-planes manually.

MitoTracker staining of embryonic mitochondria

A method adapted from Rand and colleagues was used to permeabilise embryos to molecules under 995 Da (such as Mitotracker) (Rand et al., 2010). Early embryos (0-2 hours after egg laying at 25°C) were dechorionated as for live imaging, then washed for 4 minutes in a 1:10 dilution of Citrasolv (Citrasolv, Danbury, Connecticut) in distilled water. The embryos were rinsed thoroughly with phosphate-buffered saline (PBS), and incubated in 1 M Mitotracker Red (Molecular Probes) in PBS for 5 minutes. They were again rinsed in PBS, blotted dry and mounted in halocarbon oil in a well between two coverslips on an oxygen-permeable membrane slide (Lumox, IVSS), and covered with a coverslip. They were left to develop to cellularisation in a humidified chamber at 25°C, and imaged using a 100× NA=1.4 oil-immersion objective lens.

Embryo fixation and immunostainings

Embryos were dechorionated as for live imaging, then transferred into heptane and fixed at a heptane/fixative interface for the time indicated below. Two different fixation and devitellinisation protocols were used: a 5-minute fixation in 37-41% formaldehyde solution (Fisher Scientific) with manual devitellinisation for stainings against components of the cell cortex, and an 18-minute fixation in 7.4% formaldehyde (as above, diluted fivefold in PBS) with methanol devitellinisation for all the others.

Manual devitellinisation: embryos were removed from the fixative into a basket and rinsed thoroughly with PBS with 0.1% Triton X-100 in PBS (PTX) and then PBS. They were blotted dry, stuck onto double-sided tape and immersed in PBS, and then nicked with a needle to remove the vitelline membrane. The devitellinised embryos were transferred into fresh PTX, and rinsed several times before blocking.

Methanol devitellinisation: the aqueous phase containing the fixative was removed and replaced with methanol, and the vial vortexed briefly. The devitellinised embryos sank, and

were removed to clean methanol. They were rinsed several times in methanol, rehydrated in 50% methanol: 50% water for 15 minutes, and then washed three times for 15 minutes in PTX.

Embryos were then blocked in PTX with 1% bovine serum albumin (PTB) for 30 minutes, before being incubated overnight at 4°C in primary antibody, diluted in PTB to the appropriate concentration. They were washed three times for 15 minutes in PTX, then incubated for 1 hour with fluorophore-conjugated secondary antibodies diluted 1:500 in PTB. They were washed a further three times in PTX, and mounted in Vectashield (Vector Laboratories).

A variation of this protocol was used in the case of the biotin-conjugated anti-rabbit secondary antibody: this was used at a dilution of 1:200 in PTB, and following PTX washes the embryos were incubated with streptavidin-conjugated Alexa-405 (1:50 in PTB) for 30 minutes before three further washes in PTX, and then mounted.

Staining with DAPI was carried out after washing out the secondary antibodies, by exposure to 2 g/ml DAPI in PBS for 20 minutes. Embryos were then washed in PBS for a further 15 minutes before mounting.

Antibodies

Antibodies obtained from the Developmental Studies Hybridoma Bank (NICHD and NIH, University of Iowa) were anti-Lamin (ADL84.12) and anti-DE-Cadherin (DCAD2). Other antibodies were anti-Fibrillarin (ab5821, AbCam), anti-HDEL (2E7, Sigma-Aldrich), anti- tubulin (T5667, clone GTU-88, Sigma-Aldrich) and anti-phosphotyrosine (P-Tyr-100 #9411, Cell Signaling). For staining against the Venus YFP in the CPTI lines, anti-GFP antibodies were used (rabbit ab6556, AbCam; FITC-conjugated goat ab6662, AbCam). Secondary antibodies were conjugated to Alexa fluorophores.

Imaging of immunostained embryos

Embryos of the desired stage were selected under a dissecting microscope and mounted individually in Vectashield (Vector Laboratories) in a well between two single layers of tape. Embryos were positioned ventral side up and covered with a coverslip, and were imaged on a Nikon TE-2000E microscope with a Nikon D-eclipse C1 detector, using a 60× NA=1.2 water-immersion objective, and laser illumination at 408 nm for excitation of DAPI or Alexa-405 fluorophores, 488 nm for Alexa-488 or FITC, and 543nm for Alexa-594. Interval between *z*-planes was 0.5 μm.

Superresolution imaging

Images in Fig. 5E were taken on a DeltaVision OMX V3 (Applied Precision), a 3D-SIM

(structured illumination microscope) (Gustafsson et al., 2008) system equipped with EMCCD cameras (Cascades from Photometrics). Embryos were fixed using the 5 minutes fixation protocol with manual devitellinisation. Native YFP fluorescence from sidekick-YFP and Alexa-594 for E-Cadherin staining were excited with 488 nm and 593 nm laser illumination, respectively. Images were captured with an Olympus 100× NA=1.4 oil objective, using a step size of 0.125 μm over a total depth of 4 μm. Raw data (3 angle, 5 phase) were reconstructed using softWoRx 4.5.0 software (Applied Precision).

Image processing

Maximum or average intensity projections were performed using Volocity software (PerkinElmer) or ImageJ. Annotations to movies were made in ImageJ.

The PureDenoise plugin for ImageJ was used to remove Gaussian noise from Fig. 2K (Luisier et al., 2010). Photoshop (Adobe) was used to adjust the contrast of the images.

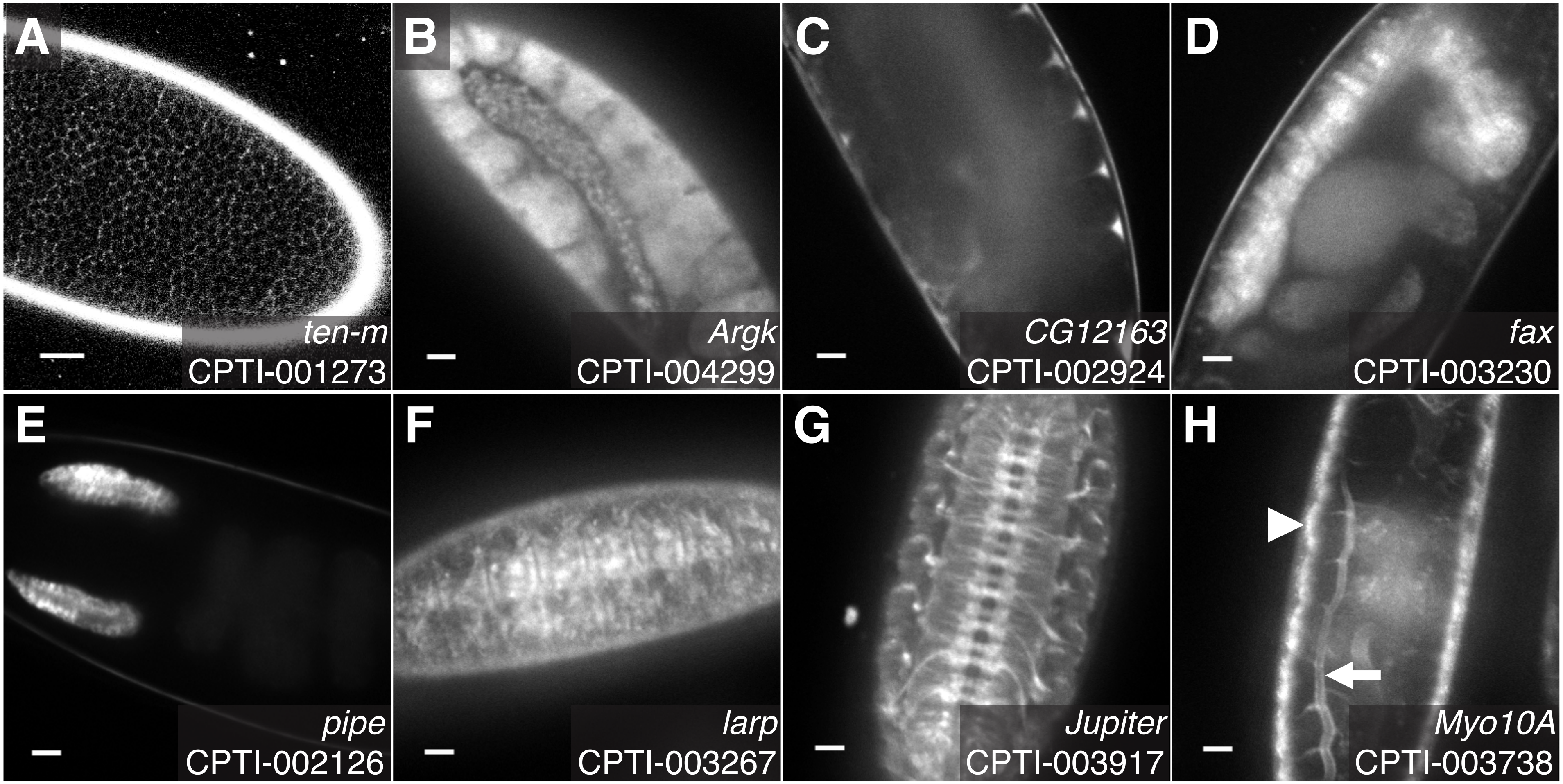

Fig. S1: Notable expression patterns of CPTI lines.

A) Metameric modulation of *tenascin-major* (*ten-m*) at stage 5 and of B) *arginine kinase (Argk)* at stage 11. C) Perivitelline expression of *CG12163* at stage 11 indicating that the YFP-tagged protein is likely to be secreted extracellularly. In stage 15 or later embryos, expression of D) *failed axon connections* (*fax*) in the central nervous system, E) *pipe* in the salivary glands, F) *La related protein* (*larp)* in muscles, G) *Jupiter* in the central and peripheral nervous system, and H) *unconventional myosin class XV* (*Myo10A*) in the epidermis (arrowhead) and the tracheal system (arrow).

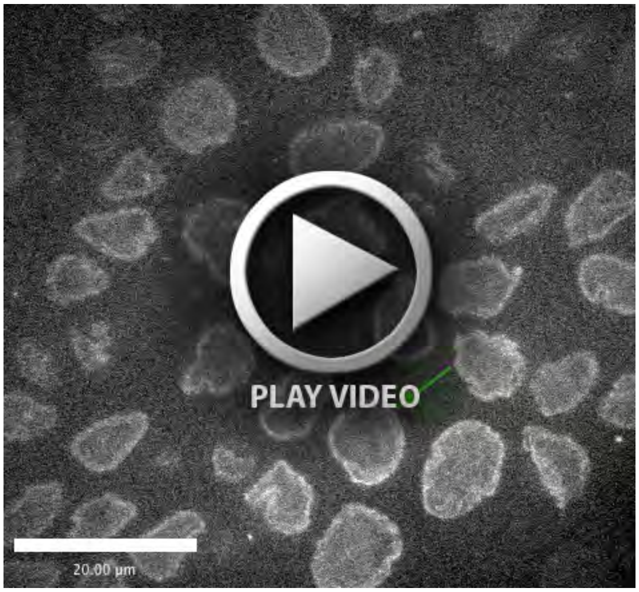

Movie 1. Dynamics of the nuclear envelope revealed by Mtor-YFP (CPTI-001044).

The irregular and dynamic shape of the nuclear envelope is visible during morphogenesis. A ventral view of the embryo during stage 8 is shown, with the cells of the ventral midline dividing as they migrate posteriorward (towards the top left) during germband extension. One cell division event is highlighted by the green arrow (for the mother cell) becoming two arrows (for the daughter cells). The nuclear envelope localisation breaks down as cytokinesis begins, but the bulk of the signal remains associated with the spindle during anaphase. Faint signal then fills the cytoplasm, with the daughter cells' nuclear envelopes reforming shortly after.

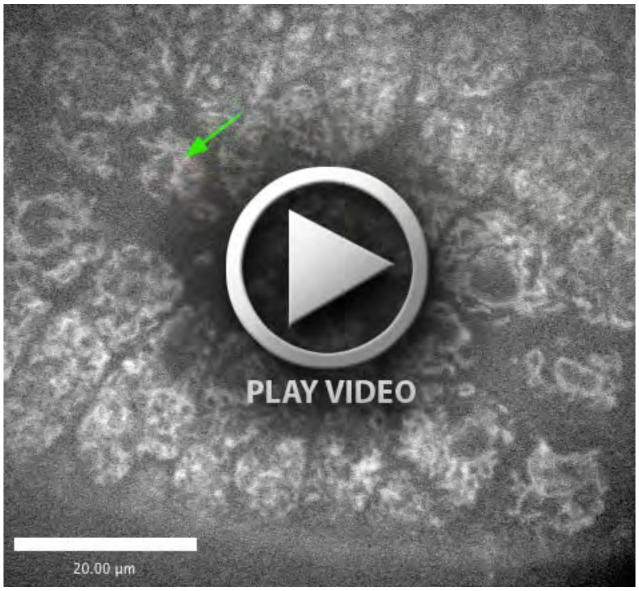

Movie 2: Dynamics of the endoplasmic reticulum revealed by l(1)G0320-YFP (CPTI-000633).

A ventral view of the embryo during stage 8 is shown, with the cells of the ventral midline dividing as they migrate posteriorward (towards the bottom right) during germband extension. The convoluted folds and perinuclear distribution that are characteristic of the ER are clearly visible. One cell division event is highlighted by the green arrow (for the mother cell) becoming two arrows (for the daughter cells). The perinuclear localisation is maintained throughout cytokinesis, in contrast to that of the nuclear envelope (see Movie 1).

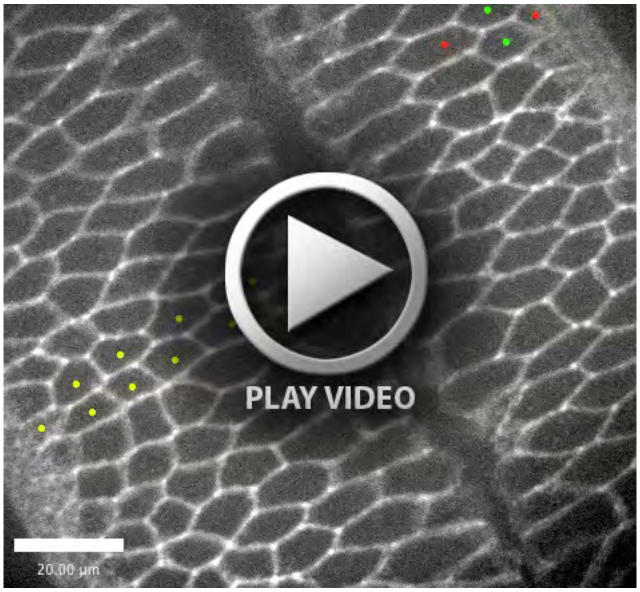

Movie 3. Cell membranes during axis extension labeled by gish-YFP (CPTI-004113).

A ventral view of the embryo during stage 8 shows the cells migrating posteriorward (towards the bottom right) during germband extension. The ventral furrow runs diagonally in the field of view. Four cells intercalating are labeled by red and green spots. A T1-T2-T3 transition occurs, removing the junction between the two green-labeled cells, then a new junction forms between the two red-labeled cells. Another intercalation event, involving nine cells that draw together to form a rosette, is highlighted with yellow dots. At the end of the movie, cell divisions can be seen in the cells of the ventral midline.

Table S1: Overview of the localisation and expression of CPTI lines.

The gene names and abbreviations (symbols) for each line are according Flybase and correspond to a unique Flybase identifier which is also listed (FBid). The chromosome and genomic location of the insertion from Flyprot is indicated when known. The note “unlocated” indicates that no sequence data is available for a given insertion, although for a subset the insertion has been linked to a chromosome. The protein-trap column indicates if there is reasonable evidence that a given line is a genuine protein-trap (Y) or if the evidence is missing or ambiguous (N). The phenotype indicates if the line is homozygous viable or lethal. All the above columns are replicated in the other supplementary tables.

The gross subcellular localisation at blastoderm cellularisation (stage 5) in either the nucleus, cytoplasm, membrane or cortex and extracellular space is indicated by a +. When localisation is found in two compartments, the brightest localisation is indicated as ++. Expression is indicated as detectable (Y) or not (N), for three embryonic stages: stage 5, 11 and late (stage 15-17). Unless specified, the notable expression patterns mentioned are for the late embryonic stages (stage 15-17). Metameric patterns are noted only for stage 5 and 11. CNS: central nervous system, PNS: peripheral nervous system. All columns have a fully searchable drop-down menu.

Download Table S1

Table S2: Nuclear localisation of the CPTI lines.

130 CPTI lines localise to the nucleus at cellularisation (stage 5). All lines but three localise to the nucleoplasm, noted as a + in the corresponding column. A subset of these lines show an enrichment in either the nucleolus, the nuclear envelope or in punctate structures, noted as ++ in the corresponding columns. For three lines, the localisation is exclusively in the nuclear envelope or in apical puncta, noted as a + in the corresponding columns. In the last column is listed additional localisation to other compartments such as the cytoplasm or the cell membrane (see Sup. Table 3 and 4 respectively). All columns have a fully searchable drop-down menu.

Download Table S2

Table S3: Cytoplasmic localisation of the CPTI lines.

258 CPTI lines localise to the cytoplasm at cellularisation (stage 5). The majority of lines localise to the cytosol without any discrete enrichment. Discrete localisation to the following recognizable organelles is listed: Endoplasmic reticulum (ER), mitochondria, centrosomes and microtubules. When a line localises at two different compartments or organelles, the localisation with brightest signal is indicated as ++. In the punctate category, 6 lines show an enrichment of the puncta in the basal end of the cell. The last column shows additional localisation to other compartments such as the nucleus or the plasma membrane (see Sup. Table 2 and 4 respectively). All columns have a fully searchable drop-down menu.

Download Table S3

Table S4: Cortical, membranous and extracellular localisations of the CPTI lines.

102 lines are listed in this table, with details of the localisation indicated for stage 5 (cellularisation) and stages 6 to 10, including position in the apico-basal axis, presence in the extracellular space, or localisation at tricellular vertices. For lines localising at more than one place, the highest number of + indicates the localisation with the brightest signal. The penultimate column lists additional localisation at stage 5 to the nucleus or the cytoplasm (see Sup. Table 2 and 3 respectively). The last column notes additional features for stage 6 to 10 such as cytoplasmic localisation, presence of an apical cap or categorised localisations during cytokinesis (see Figure 6). All columns have a fully searchable drop-down menu

Download Table S4
